# Advances on Graphyne‐Family Members for Superior Photocatalytic Behavior

**DOI:** 10.1002/advs.202003900

**Published:** 2021-03-11

**Authors:** André Torres‐Pinto, Cláudia G. Silva, Joaquim L. Faria, Adrián M. T. Silva

**Affiliations:** ^1^ Laboratory of Separation and Reaction Engineering—Laboratory of Catalysis and Materials (LSRE‐LCM) Faculdade de Engenharia Universidade do Porto Rua Dr. Roberto Frias Porto 4200‐465 Portugal

**Keywords:** carbon materials, hydrogen production, oxygen evolution, photocatalysis, water treatment

## Abstract

Graphyne (GY) and graphdiyne (GDY) have been employed in photocatalysis since 2012, presenting intriguing electronic and optical properties, such as high electron mobility and intrinsic bandgap due to their high *π*‐conjugated structures. Authors are reporting the enhanced photocatalytic efficiency of these carbon allotropes when combined with different metal oxides or other carbon materials. However, the synthesis of graphyne‐family members (GFMs) is still very recent, and not much is known about the true potential of these photocatalytic materials. In this review article, the implications of different synthesis routes on the structural features and photocatalytic properties of these materials are elucidated. The application of GFMs in the nicotinamide adenine dinucleotide (NADH) regeneration, hydrogen and oxygen evolution, and carbon dioxide reduction is discussed, as well as in the degradation of pollutants and bacteria inactivation in water and wastewater treatment.

## Graphical abstract

This literature review of graphyne‐family members for heterogeneous photocatalysis is a basis to extend the knowledge about these structures and their respective photocatalytic applications. The synthesis routes and photoactivity results are discussed considering the most relevant data for the development of efficient and sustainable systems for the world of tomorrow.

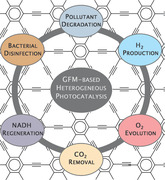

## 1 Introduction

Graphyne‐family members (GFMs) are a series of 2D carbon allotropes, consisting of one‐atom‐thickness sheets topologically similar to graphene (GR) and graphenederivatives (GRDs).^[^ ^1^ ^]^ GFMs present a unique atom arrangement with *sp*‐ and *sp* ^2^‐hybridizations of carbon bonds periodically distributed in a 2D planar honeycomb‐like structure. The acetylenic (—C≡C—) and/or butadiyne (—C≡C—C≡C—) bonds in GFMs replace some (or all) the single carbon bonds (—C—C—) characteristic of GRDs, conferring distinct electronic and optical properties. According to the number (one or two) of triple bonds between adjacent benzene rings, these materials are classified as graphynes (GY—one triple bond) or graphdiynes (GDY—two triple bonds).

The history of GFMs began in 1987, with the envisioning of 2D *sp*‐ and *sp* ^2^‐hybridized carbon planes by Baughman et al., through computational chemistry methods for describing different GFM structures and respective heat formation energies.^[^ ^2^ ^]^ Since then, much effort has been put in finding new routes for the production of this family of materials. GDY annulenic substructures (i.e., macrocycles) were experimentally prepared by Haley's group via oxidative cyclooligomerization of polyynes.^[^ ^3^, ^4^, ^5^ ^]^ However, until 2010, the majority of publications were focused on computational calculation methods to describe hypothesized carbon allotropes. Density functional theory (DFT) calculations were applied by different research groups, such as those from Baughman and Ivanovskii, to characterize and discuss the properties of likewise theoretical GFM nanotubular structures.^[^ ^6^, ^7^, ^8^, ^9^, ^10^, ^11^ ^]^

In 2010, the synthesis of *γ*‐GDY was reported for the first time by Li et al. through a cross‐coupling reaction on a copper substrate.^[^ ^12^ ^]^ This work represents a breakthrough in this field since no other material from this family (GFMs) had been synthesized before. Since then, researchers have developed new synthesis methods to produce different GFMs. Undoubtedly, since this discovery, the go‐to synthesis route for GFMs is the denominated Glaser coupling reaction.^[^ ^12^ ^]^ Briefly, this method consists on treating [(trimethylsilyl)ethynyl]zinc chloride on tetrahydrofuran (THF) solution with tetrakis(triphenylphosphine)‐palladium(0) and toluene for 3 d at high temperature, then in acidic conditions extract the organic layer by continuous washing and chromatography. Afterward, the resulting product would be mixed with tetra‐*n*‐butylammonium fluoride (TBAF) on a THF solution, use vacuum to remove the solvent and redilute it in pyridine to slowly deposit it on copper foils under an inert atmosphere, where it remained for 2 d, being finally washed with acetone and *N*,*N*‐dimethylformamide (DMF).^[^ ^12^ ^]^ This procedure requires large amounts of relatively toxic solvents and expenditures related to the specific operating conditions that brand this process as economically and environmentally unfeasible.

**Figure 1:**
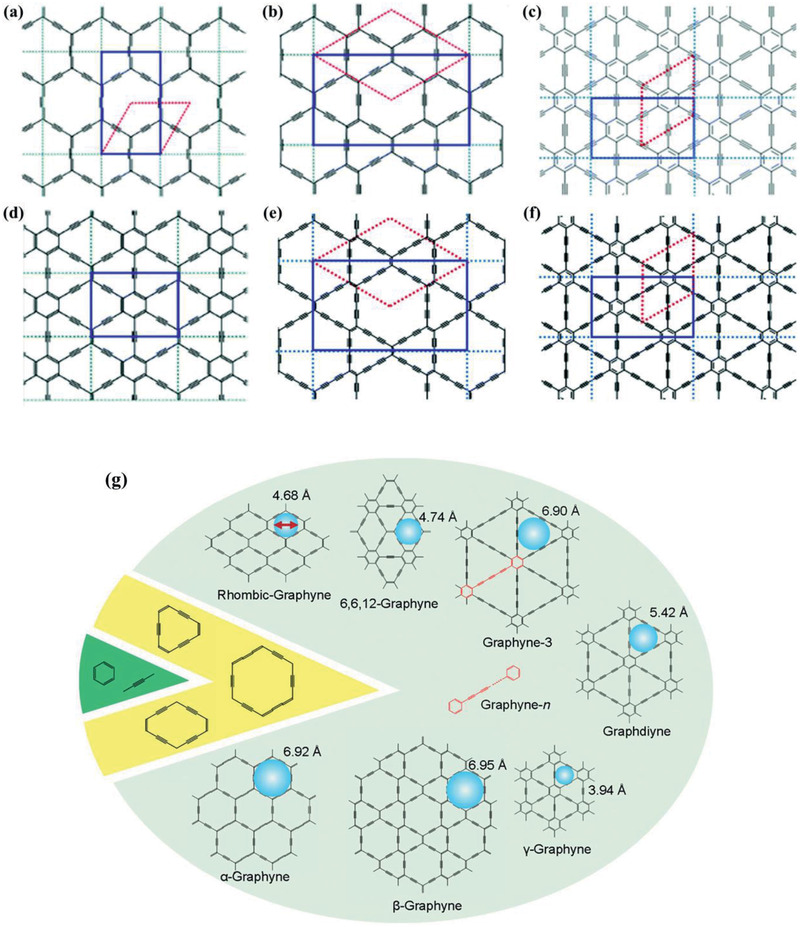
Structural configuration of different GFMs (highlighting their fundamental units in color): a) *α*‐GY, b) *β*‐GY, c) *γ*‐GY, d) 6,6,12‐GY, e) *β*‐GDY, f) *γ*‐GDY, and g) portraying besides the aforementioned structures, a rhombic‐GY, the building blocks of the ‐yne structures and their nanopore diameters. a–f) Reproduced with permission.^[^ ^18^ ^]^ Copyright 2020, Royal Society of Chemistry. g) Reproduced with permission.^[^ ^26^ ^]^. Copyright 2019, Wiley‐VCH.

Based on the in situ growth method reported by Li et al., many studies apply other wet chemistry routes to prepare GFMs with a large surface area and to keep a reasonable control on the structure of these materials.^[^ ^12^ ^]^ Some of those include the growth of GDY films on specific substrates, liquid–liquid or liquid–vapor interfaces.^[^ ^13^, ^14^, ^15^, ^16^, ^17^ ^]^ However, these procedures have certain disadvantages such as low yields and the need for solvents (tetrahydrofuran, toluene, pyridine, among others), metal substrates and inert conditions.^[^ ^12^ ^]^ Thus, many authors have been focusing their attention on alternative methods, to fight the low‐scalability and poor sustainability of these routes.^[^ ^18^, ^19^, ^20^, ^21^, ^22^, ^23^, ^24^, ^25^ ^]^ Chemical vapor deposition and ultrahigh vacuum are regarded as effective routes to reduce solvent usage.^[^ ^20^, ^21^ ^]^ Microexfoliation (based on an explosion method) was proposed owing to an ultrafast and direct heating procedure.^[^ ^22^ ^]^ Mechanochemistry is a simple, yet a recent, solution to conduct sustainable experimental assays through earth‐abundant precursors and using accessible ball‐milling apparatus.^[^ ^23^, ^24^, ^25^ ^]^ All these different fabrication methods prompt the design of several GFMs, shown in **Figure** **1**, that can be defined as *α*‐GY, *β*‐GY, 6,6,12‐GY, *γ*‐GY, rhombic‐GY, *β*‐GDY, *γ*‐GDY, among others, depending on the respective structure and molecular distribution.^[^ ^18^, ^26^ ^]^

Computational and experimental studies have both defended that the unique structure of GFMs can provide a facile control of the surface chemistry, textural properties and high electronic mobility, yet with prone hydrophobicity.^[^ ^1^, ^18^ ^]^ Some modifications to increase hydrophilicity include the combination with other materials, oxidation or structural tuning. In particular, metal‐free GFM‐based photocatalysts are attracting increasing interest since they have been described as extremely efficient in both computational.^[^ ^27^, ^28^, ^29^, ^30^ ^]^ But also in and experimental studies, as detailed in this review.^[^ ^31^, ^32^, ^33^, ^34^, ^35^, ^36^, ^37^, ^38^, ^39^, ^40^, ^41^, ^42^, ^43^, ^44^, ^45^, ^46^ ^]^ Theoretical studies reveal that GFM materials have electronic and optical properties similar to or superior to graphene.^[^ ^18^ ^]^ They show semiconducting properties, which means they possess a direct bandgap and carrier mobility comparable to that of graphene, both being relevant properties for photocatalytic applications.^[^ ^47^, ^48^, ^49^ ^]^ For instance, GDY was described applying first‐principle DFT calculations, as a semiconducting catalyst with a charge carrier mobility reaching 2 × 10^5^ cm^2^ V^–1^ s^–1^ (i.e., similar to graphene).^[^ ^50^, ^51^ ^]^ These characteristics and the high *π*‐conjugation of GFM networks have attracted interest to apply these materials in energy storage and electrochemical sensing, besides electrocatalytic and photocatalytic applications.^[^ ^52^, ^53^, ^54^, ^55^ ^]^

The application of GFMs in the literature is limited since the first experimental study is relatively recent and because there is still limited information on easy and sustainable synthetic routes. More experimental studies have been reported so far for electrocatalysis and overall water splitting for hydrogen production as well as oxygen evolution.^[^ ^56^, ^57^, ^58^, ^59^, ^60^, ^61^, ^62^, ^63^ ^]^ Notably, these papers are interesting to address as their conclusions bridge electrocatalysis with its electronic transfer proficiency and photoinduced reactions that benefit from elevated charge carrier mobility. They introduce GFMs as a resilient matrix to promote a strong interaction with metal‐based semiconductors, considering advanced characterization data and water splitting results. Moreover, these publications explain the mechanistic pathways for redox reactions. The electronic charge transfer is investigated more in‐depth. We can state that GFM heterostructures stabilize the charge carriers on the conduction bands energy levels with the help of the *sp* ^2^‐hybridized carbon planes of GFMs, driven by the excitation of electrons in the other moiety of the hybrid materials.

Despite being very recent, many factors are still impeding the diverging applications of GFMs as suitable catalysts. Their synthesis routes need to be tuned to properly instigate the specific electronic transitions in catalytic reactions. Furthermore, a substantial barrier to photocatalysis usage is the need to conjugate GFMs with a semiconducting material to create an electron differential and not constrain charge accumulation, as mainly justified by theoretical studies.^[^ ^64^ ^]^

**Figure 2:**
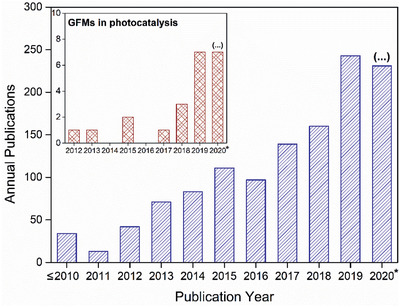
Publication interest in GFMs: number of scientific articles published yearly involving “graphyne” or “graphdiyne.” Inset: publications reporting experimental results of GFMs for photocatalytic applications. Data queried from Scopus as of 2020 October 4.

Most scientific articles dealing with GFMs focus on theoretical studies, as was reported in a recent review paper.^[^ ^64^ ^]^ However, that tendency is changing over time with more experimental reports being published each year. In fact, after the first effective experimental synthesis of these materials in 2010, a nearly exponential evolution was registered, reaching 247 articles in 2019 (**Figure** **2**), and the number of publications is therefore expected to continue rising.^[^ ^12^ ^]^ A systematic revision on experimental research of GFMs was performed to refine the first round of results, now focusing specifically on “photocatalysis” (inset of Figure 2). After the first report published in 2012, the amount of experimental studies employing GFMs for photocatalytic applications is quickly advancing with 22 experimental papers, whereas only 19 are solely computational works.

There are already a few review articles concerning the synthesis and application of GFM materials.^[^ ^65^, ^66^, ^67^, ^68^ ^]^ In specific, one provides relevant information about the use of *γ*‐GDY as a support for electrocatalytic pollutant degradation and hydrogen production; and another is a recent mini review illustrating the evolution of synthesis routes from small alkyne structures into 2D and 3D GFMs.^[^ ^31^, ^68^ ^]^ However, agreeing with the brief history of GFMs, there was not a review paper exclusively focused on the development of GFM photocatalytic materials. The development of GFM‐based semiconductor composites is a recent challenge and, therefore, there is very limited information in the literature regarding on how to properly modulate GFMs for enhanced photoactivity with economical and sustainable feasibility.

Finally, with this review, we intend to collect important information to facilitate further studies on GFM‐hybrid photocatalysts in order to improve their performance in a wide range of photocatalytic applications. It is crucial to think on a global scale when preparing these nanoscale materials, in regards not only to fabrication costs but also environmentally friendly routes. Moreover, to add to this, it is imperative to arrange a way to compare different GFM materials that are employed in many applications, to stop focusing on small synthesis details and begin preparing efficient, green and cost‐effective systems. Altogether, we hope that this systematic literature review allows for the efficient and sustainable development of GFM photocatalysts and improve their application.

## 2 GFMs Application in Photocatalysis

Photocatalysis has become an impactful technology with a broad range of applications which employs light as an energy source for the activation of the photocatalysts. This review is mainly focused on *γ‐*GY and *γ‐*GDY (hereafter referred to GY and GDY), which are the main GFMs effectively synthesized and experimentally tested in photocatalytic applications.^[^ ^32^, ^33^, ^34^, ^35^, ^36^, ^37^, ^38^, ^39^, ^40^, ^41^, ^42^, ^43^, ^44^, ^45^, ^46^, ^69^, ^70^, ^71^ ^]^ GY and GDY also represent the structures of GFMs with predicted highest stability due to their lower heat of formation energies.^[^ ^2^, ^3^ ^]^ Besides the *γ‐*structures, some publications report the fabrication of GDY materials with different configurations based on their precursors and synthesis method, namely *β‐*GDY and even amorphous GDY.^[^ ^55^, ^72^, ^73^ ^]^ Also, the synthesis and application of 3D GFMs will be discussed in a following section.^[^ ^74^ ^]^

**Figure 3:**
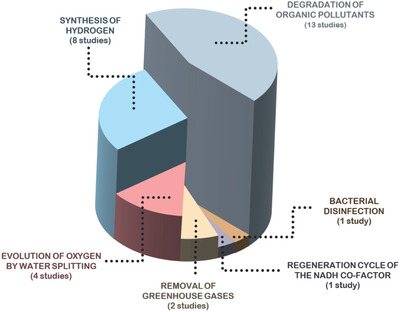
Distribution of the photocatalytic experimental studies of GFM‐based materials. Data retrieved from Scopus, from keywords (“graphyne” or “graphdiyne” and “photocatal*”), and updated as of 2020 October 4.

In total, 22 scientific articles were found with the aim to experimentally employ GFM materials in photocatalysis, which are distributed in **Figure** **3** taking into consideration the different photocatalytic applications. Degradation of pollutants, H_2_ production and O_2_ evolution are the three major applications of GFM‐based photocatalysts, representing 38%, 28%, and 19% of the studies, respectively. Many studies present a comparison with metal oxides, for instance, TiO_2_ is many times selected for being known to be a stable and inexpensive photocatalyst with an appropriate band structure for several applications, and because it has relatively low toxicity; however, one limitation is the fast electron/hole recombination.^[^ ^75^ ^]^

The following chapters on this review discuss the major findings on the use of GFM‐based materials in specific photocatalytic applications: 1) degradation of organic pollutants and bacterial disinfection in liquid phase; 2) hydrogen production; 3) oxygen evolution by water splitting; 4) removal of greenhouse gases; and 5) the regeneration cycle of the nicotinamide adenine dinucleotide (NADH) co‐factor. The reaction conditions and main results of photocatalytic activity are summarized in **Table** **1**, and when authors applied the same GFM material toward distinct objectives, they were accounted for in the distribution on Figure **3** and disclosed in Table 1.

**Table 1:** GFM‐based materials in photocatalytic studies

Material	Application	Reactant solution	Light source	Activity	Reference photocatalyst (RP)	RP activity	RP improvement factor	Ref.
*β‐*GDY/TiO_2_	MB degradation	30 mg_cat_; 40 mL aq. solution: 0.01 g L^–1^ (0.47 μmol L^–1^) MB	500 W Xe lamp (60 µW cm^2^)	100% rem. (30 min)	TiO_2_	80% rem. (30 min)	1.25	^[^ ^55^ ^]^
	MB degradation				TiO_2_‐GR	90% rem. (30 min)	1.11	
GDY/TiO_2_	MB degradation	30mg_cat_; 40 mL aq. solution; 0.01 g L^1^ (0.27 μmol L^–1^) MB	100 mW cm^–2^ Xe lamp	0.0247 min^–1^	TiO_2_	0.0152 min^–1^	1.62	^[^ ^41^ ^]^
GDY/APO emulsion	MB degradation	7 mL oil:water (1:0.4) emulsion; 5 mmol L^1^ MB	500 W Xe lamp	0.477 min^–1^	APO	0.067 min^–1^	7.1	^[^ ^62^ ^]^
GDY/Ag/AgBr/GO	MO degradation	9 mg_cat_; 9 mL aq. solution; 60 mg L^–1^ MO	500 W Xe lamp (*λ* > 400 nm)	0.098 min^–1^	Ag/AgBr	0.011 min^–1^	8.91	^[^ ^42^ ^]^
GDY/ZnO	MB degradation	0.5 mg_cat_; 100 mL aq. solution; 1 μmol L^1^ MB	UV light	0.00426 min^1^	ZnO	0.00181 min^1^	2.4	^[^ ^64^ ^]^
	RhB degradation			0.00298 min^1^	ZnO	0.00166 min^1^	1.8	
GDY/N‐TiO_2_	RhB degradation	25 mg_cat_; 40 mL aq. solution; 10 mg L^–1^ RhB	500 W Xe lamp (*λ* > 420 nm)	90% RhB rem. (240 min)	N‐TiO_2_	78% RhB rem. (240 min)	1.15	^[^ ^44^ ^]^
	TC degradation	25 mg_cat_; 40 mL aq. solution; 5 mg L^1^ TC		77% TC rem. (240 min)		64% TC rem. (240 min)	1.20	
GY/APO	NFL degradation	50 mg_cat_; 100 mL aq. solution; 20 mg L^–1^ pollutant	300 W Xe lamp (*λ* > 420 nm)	0.798 min^–1^	APO	0.052 min^–1^	15.3	^[^ ^45^ ^]^
	HNP degradation			1.072 min^–1^		0.111 min^–1^	9.6	
	PH degradation			0.415 min^–^1; 100% PH rem. (16 min)		0.021 min^–1^; 32% PH rem. (20 min)	19.7	
*β*‐GDY/TiO_2_	MB degradation	20 mg_cat_; 40 mL aq. solution; 0.01 g L^1^ (0.27 μmol L^–1^) MB	100 mW cm^2^ Xe lamp (*λ* > 300 nm)	90% MB rem. (30 min)	TiO_2_	70% MB rem. (30 min)	1.3	^[^ ^68^ ^]^
*γ*‐GDY/TiO_2_				80% MB rem. (30 min)			1.1	
TA‐GY	MO degradation	2 g_cat_ L^–1^; 30 mL aq. solution; 30 mg L^1^ pollutant	500 W Xe lamp (150 mW cm^2^)	99% MO rem. (8 h)	–	–	–	^[^ ^46^ ^]^
	*E. coli* inactivation	Lysogeny Broths medium with *E. coli*	500 W Xe lamp (100 mW cm^2^)	100% *E. coli* inactivation (1 h)	–	–	–	
GDY/CdS	H_2_ production	2 mg_cat_; 5 mL aq. solution; 0.3 mol L^–1^ TEOA	200 mW cm^–2^ LED (*λ* = 450 nm)	4.1 mmol g^–1^	CdS	1.6 mmol g^–1^	2.6	^[^ ^63^ ^]^
GY/TiO_2_	H_2_ production	20 mg_cat_; 100 mL water:methanol 1:9 solution	300 W Xe lamp	77.6 μmol (4 h)	TiO_2_	9.26 μmol (4 h)	8.4	^[^ ^38^ ^]^
GDY/GCN	H_2_ production	80 mL aq. solution; 50 mg_cat_; 15% TEOA; 1% Pt co‐catalyst	350 W Xe lamp (*λ* > 420 nm)	39.6 μmol h^1^	GCN	5.9 μmol h^–1^	6.7	^[^ ^39^ ^]^
Amorphous GDY	H_2_ production	16 mL water:acetonitrile 1:1 solution; 10 mg_cat_; 2 mL TEOA; Pt co‐catalyst	300 W Xe lamp (*λ* > 395 nm)	972 μmol h^–1^ g^–1^	GDY	490 μmol h^–1^ g^–1^	2.0	^[^ ^43^ ^]^
GDY/CuI	H_2_ production	10 mg_cat_; 20 mL TEOA (15% v/v); Pt co‐catalyst;	5 W LED	93.2 μmol h^–1^	GDY	5.9 μmol h^–1^	15.8	^[^ ^40^ ^]^
					CuI	31.3 μmol h^–1^	3.0	
DBA‐GDY	H_2_ production	20 mg_cat_; 30 mL TEOA (15% v/v); 0.5 wt.% Pt co‐catalyst	300 W Xe lamp (*λ* > 420 nm)	340 μmol h^–1^ g^–1^	–	–	–	^[^ ^69^ ^]^
MoSe_2_/TiO_2_/GY	H_2_ production	20 mg_cat_;100 mL water:methanol 1:9 solution	300 W Xe lamp	800 μmol h^1^ g^–1^	MoSe_2_/TiO_2_	250 μmol h^1^ g^–1^	3.2	^[^ ^66^ ^]^
					TiO_2_	129 μmol h^1^ g^–1^	6.2	
GCN/GDY	H_2_ production	20 mg_cat_; 80 mL TEOA (15% v/v); Pt co‐catalyst	300 W Xe lamp (*λ* > 400 nm)	22 712 μmol h^–1^ g^–1^	GCN	7341 μmol h^–1^ g^–1^	3.1	^[^ ^67^ ^]^
GDY/GCN/APO	O_2_ evolution	80 mL aq. solution: 10 mg_cat_	300 W Xe lamp (*λ* > 420 nm)	753.1 μmol g^–1^ h^–1^	APO	61.4 μmol g^–1^ h^–1^	12.2	^[^ ^33^ ^]^
GDY/APO emulsion	O_2_ evolution	37.5 mL emulsion; 15 mL APO	500 W Xe lamp	3.5 mg L^–1^ (50 min)	APO	0.5 mg L^–1^ (50 min)	7.0	^[^ ^62^ ^]^
GDYO	O_2_ evolution	10 mg_cat_; 50 mL aq. solution; 0.01 mol L^1^ AgNO_3_	300 W Xe lamp (*λ* > 420 nm)	150.7 μmol g^–1^ h^–1^	GDY	4.8 μmol g^–1^ h^–1^	31.4	^[^ ^35^ ^]^
CdS/GY	CO_2_ reduction	20 mg_cat_; 10 mL H_2_SO_4_ aq. solution (2 mol L^–1^) saturated with NaHCO_3_	350 W Xe lamp	18.7 μmol g^–1^ h^–1^	CdS/GO	15.0 μmol g^–1^ h^–1^	1.25	^[^ ^65^ ^]^
GDY/TiO_2_	CO_2_ reduction	10 mg_cat_; 30 mL water:acetonitrile 1:30 solution	350 W Xe lamp	50.5 μmol_CO_ g^–1^ h^–1^	TiO_2_	15.8 μmol_CO_ g^1^ h^–1^	3.2	^[^ ^36^ ^]^
	O_2_ evolution (simultaneous)			30.9 μmol g^–1^ h^–1^	TiO_2_	8.3 μmol g^1^ h^–1^	3.7	
N‐GDY	NADH regeneration	0.30–1.50 mg_cat_ mL^–1^; 0.17 mmol L^–1^ [Cp*Rh(bpy)(H)]^+^; 0.67 mmol L^–1^ NAD^+^	300 W Xe lamp (*λ* > 420 nm)	35% regeneration (3 h)	N/A	N/A	N/A	^[^ ^61^ ^]^

GDY: graphdiyne; GY: graphyne (in the absence of Greek letter, the structure is *γ*‐GDY and *γ*‐GY); APO: silver phosphate; GCN: graphitic carbon nitride; DBA‐GDY: dehydrobenzoannulene‐based GDY; TA GY: triazine based GY; MB: methylene blue; MO: methyl orange; RhB: rhodamine B; NFL: norfloxacin; HNP: 2‐hydroxynaphtalene; PH: phenol; aq.: aqueous; TEOA: triethanolamine; rem.: removal.

### 2.1 Water Treatment

Water treatment is of utmost importance, mostly due to restrictions with water availability and distribution worldwide.^[^ ^76^ ^]^ Pollutants commonly found in wastewaters can be resistant to conventional treatment processes, and different technologies may be needed for their removal, including advanced oxidation technologies.^[^ ^77^, ^78^ ^]^ GFMs have already been tested in a laboratory scale for the removal of different contaminants (e.g., dyes and bacteria).

Wang et al. combined TiO_2_ with different carbon materials such as GDY, carbon nanotubes (CNT) and graphene (GR).^[^ ^55^ ^]^ GDY was prepared by a cross‐coupling reaction on a copper foil, and the composites were achieved by a hydrothermal method. These composites proved to be more efficient than TiO_2_ alone in methylene blue (MB) degradation under visible light. The calculated bandgap energies of the materials were significantly lower in the presence of the carbon materials, which rationalizes the enhanced activity of the hybrid materials. The material combining TiO_2_ with GDY showed the highest photocatalytic efficiency for MB removal, followed by GR/TiO_2_, CNT/TiO_2_ and finally TiO_2_ alone.

Yang et al. synthesized a GDY/TiO_2_ material through a hydrothermal method, GDY being obtained via a cross‐coupling reaction on a copper foil.^[^ ^41^ ^]^ The composite was characterized by DFT, which suggested the existence of enhanced electronic mobility between the carbon‐titania linkages. In the photocatalytic degradation of MB, the apparent rate constant increased from 0.0152 to 0.0247 min^–1^, corresponding to an improvement factor of 1.62 (Table 1). This enhancement is explained by the electron‐accepting ability of the *π*‐conjugated system of GDY that captures the photogenerated electrons from TiO_2_.

A work performed by Zhang et al. also showed that an Ag/AgBr/GO/GDY hybrid material was more efficient for the degradation of methyl orange (MO) than Ag/AgBr/GDY (by 2.9 times) or Ag/AgBr (by 8.9 times), GO acting as a linking agent.^[^ ^42^ ^]^ The high photocatalytic efficiency increase in comparison with Ag/AgBr is believed to be owed to the high electron mobility of the *π*‐conjugated system of GDY.

Thangavel et al. produced a metal oxide‐GFM nanohybrid with GDY grown by cross‐coupling on a copper foil and combination with ZnO by a hydrothermal route.^[^ ^43^ ^]^ The GDY/ZnO composite was selected for the degradation of MB and Rhodamine B (RhB), where it achieved photodecomposition rate constants 2 times higher than those obtained with bare ZnO. Moreover, the total organic carbon (TOC) content removal increased from 35% to 45% for MB and from 25% to 55% for RhB when ZnO and GDY/ZnO were tested, respectively, under 120 min irradiation (Table 1). The authors explain the increased photoefficiency by the photoluminescence quenching ascribed to the mechanism of electron transfer from ZnO to GDY, hindering of charge carriers recombination. Also, the higher availability of electrons on the GDY structure makes it more prone to react with dissolved oxygen to generate O_2_^•−^ radicals and allows for the remaining holes in ZnO to form HO^•^ radicals. The presence of these species facilitates the photocatalytic decomposition of both dye compounds.

Pickering emulsions are widely used as an alternative method to stabilize organic‐inorganic materials for several applications.^[^ ^79^ ^]^ Guo et al. prepared a Pickering emulsion with silver phosphate (APO) and GDY by sonication, with GDY previously synthesized by a cross‐coupling reaction on a copper foil.^[^ ^34^ ^]^ The emulsion consisted of the combination of GDY and APO dispersed by sonication in a water and oil mixture. This system was used for the photocatalytic degradation of MB. As will be discussed in Section 2.3, the main application of Guo's GDY/APO Pickering emulsion is the removal of MB, but was also studied for the photocatalytic evolution of O_2_.^[^ ^34^ ^]^ GDY alone led to an almost negligible photocatalytic removal of MB, with APO alone achieved 55% MB removal in 10 min.

In contrast, the APO/GDY emulsion yielded ≈100% MB removal in the same irradiation time, whereas those prepared with APO and CNTs or GR achieving only about 75% removal in 10 min (Table 1). Briefly, the mechanism consisted on photogenerated electrons that migrate from APO to highly electroconductive GDY, which blocked charge carrier recombination and allowed for an enhanced hole‐oxidation of MB (**Figure** **11**). This mechanism is further elucidated when O_2_ evolution was investigated by these authors and is described in Section 2.3 (Figure 11).

Dong et al. prepared a composite with N‐doped TiO_2_ nanosheets and a GDY suspension by using a hydrothermal method.^[^ ^44^ ^]^ This material was tested for the photocatalytic degradation of RhB, a 90% removal being achieved after 240 min of irradiation (in contrast with pristine TiO_2_ or N‐doped TiO_2_: 37% and 78%, respectively). The photocatalytic degradation of tetracycline hydrochloride (TC) was also studied: 77%, 64%, and 43% removals being respectively obtained with the composite, NTiO_2_ and TiO_2_ after 240 min (Table 1). The enhancement is mainly attributed to the 2D *π*–*π* conjugated structure of GDY, resulting in high electron mobility and improved charge separation. The bandgap energy, calculated by diffuse reflectance spectroscopy, is succeedingly smaller for TiO_2_, N‐TiO_2_, and N‐TiO_2_/GDY, but with very slight variations. A marked difference is observed when the quenching of photoluminescence intensity for the original N‐TiO_2_ is compared with that observed for the composite, validating the rapid transfer of photogenerated electrons to GDY. The mechanism of RhB is attributed to the oxidative action of superoxide radicals (O_2_^•−^) formed by oxygen reduction and photogenerated holes (*h* ^+^).

**Figure 4:**
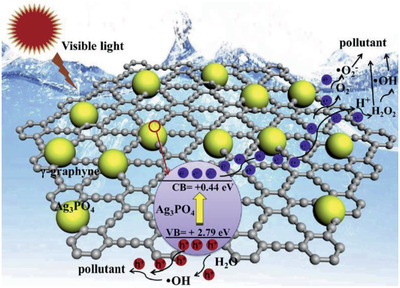
Photocatalytic mechanism for GY/APO composite under visible light irradiation. Reproduced with permission.^[^ ^45^ ^]^ Copyright 2019, Elsevier.

A mechanochemical method was also used by Lin et al. to prepare GY, which was followed by the synthesis of a composite with APO through electrostatic self‐assembly by sonication and continuous stirring.^[^ ^45^ ^]^ Lin et al. tested the photocatalytic efficiency of APO alone and the GY/APO composite material for the photocatalytic degradation of three different compounds individually: norfloxacin (NFL), 2‐hydroxynaphtalene (HNP), and phenol (PH). The composite showed better activity in all situations. The smallest improvement was observed for HNP (9.6 times faster removal rate with GY/APO in comparison with APO), while under 20 min irradiation PH degradation was 100% with the composite and only 32% with APO alone (Table 1). Coupling both materials demonstrated an extension in light absorption in the visible range, which constitutes an advantage for the application of this photocatalyst in solar‐driven processes. The Mott–Schottky plots and X‐ray photoelectron spectra of APO showed that it has a bandgap energy of 2.35 eV, with a VB of +2.79 eV (vs NHE) with strong oxidizing ability, and a corresponding CB energy of +0.44 eV (vs NHE), as shown in **Figure** **4**. The composite's band levels showed a slight shift toward more negative energies ascribed to the electronic interaction between both materials. In terms of photoluminescence, it was observed that the composite had a much lower photoluminescence intensity which could mean easier charge separation. Also, with time‐resolved photoluminescence, it was seen that after the addition of GY, the radiative lifetimes decreased significantly, which is owed to the electron transfer from APO to GY effectively achieving charge carriers recombination suppression.

The authors investigated the PH degradation mechanism using different scavenging agents, such as EDTA‐2Na (ethylenediaminetetraacetic acid disodium salt for photogenerated holes), BQ (benzoquinone for O_2_^•−^) and IPA (isopropyl alcohol for HO^•^). They concluded that O_2_^•−^ and *h* ^+^ were the primary species responsible for the photocatalytic transformations. In agreement with these experiments, the calculated band potentials are sufficient for water and oxygen to generate HO^•^ and O_2_^•−^ radicals (Figure 4). Moreover, the high electrical conductivity of GY promotes the rapid charge transfer of electrons from APO to the GY structure and inhibits charge carrier recombination. This allows for a powerful oxidizing attack from the unrestricted holes and enhances the degradation process.

Another type of GFM, *β*‐GDY was produced by Li et al. produced *β*‐GDY following the typical Glaser cross‐coupling method on a copper foil but using a tetraethynylethene precursor and different solvents.^[^ ^72^ ^]^ The progress done by these authors showed a more densely *π*‐conjugated material, in comparison with *γ*‐GDY (discussed throughout this review as GDY). Also, a shift in light absorption to higher wavelengths was observed by UV–vis spectroscopy, which may be rationalized by the larger number of —C≡C— bonds. The adjustment on the synthesis route the authors performed, compared to the typical Glaser reaction, effectively achieved a higher amount of acetylenic bonds, i.e., *β*‐GDY presented 27% fewer butadiyne linkages compared to GDY, and therefore a stronger *π*‐conjugation.^[^ ^80^ ^]^ This characteristic endows *β*‐GDY with a more efficient charge transfer, being a highly electroconductive material. At the same time, besides high electrical conductivity, which is beneficial for photocatalytic applications, *β*‐GDY presents no natural bandgap, fundamentally differing it from *γ*‐GDY. Undesirably, *β*‐GDY must be irrevocably coupled to a photoresponsive material for photocatalytic application.^[^ ^81^, ^82^ ^]^ Moreover, *β*‐GDY is more prone to establish strong interactions with metallic compounds due to its linkage framework, thus the fabrication of composites with TiO_2_ in Li's work resulted in abundant bonds between titania and acetylene linkers.^[^ ^72^ ^]^ After photoactivation of TiO_2_, electrons are believed to be easily transported to those titania‐acetylene bonds and function as charge recombination blockers, allowing for improved photocatalytic activity. Li and coworkers tested TiO_2_ alone (≈80% removal after 30 min) and two composites: GR/TiO_2_ (≈90% removal) and *β*‐GDY/TiO_2_ (100% MB removal) in the degradation of MB (Table 1). The enhanced efficiency of both composite materials was attributed to the hindering effect on electron/hole recombination. *β*‐GDY/TiO_2_ showed higher photocatalytic efficiency than GR/TiO_2_ due to the higher electron mobility distinctive of GFM materials compared to GR, which endowed the composite with faster charge transfer.

In the abovementioned studies, it is demonstrated that the removal rates of the organic compounds were improved by employing a hybrid GFM‐metal material instead of solely the metal‐based semiconductor. This was due to the enhanced electronic properties of the hybrid material stimulated by the presence of the strong —C≡C— linkage framework of GFMs. Combining these materials resulted in improved photocatalytic activity, ascribed to the excellent charge transfer abilities that allowed for slower exciton recombination rates and consecutively propelled the formation of oxidizing species. Another advantage is the maneuverability of GFMs by the fabrication route, as observed in the case of *β*‐GDY and *γ*‐GDY, promoting distinct electronic properties.

**Figure 5:**
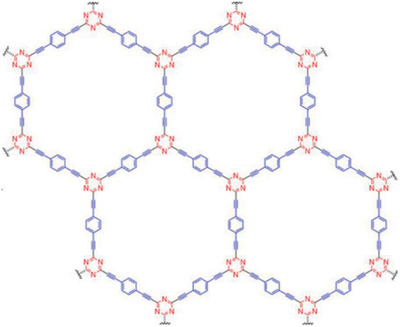
Proposed structure of the triazine‐based GFM (TA‐GDY). Reproduced with permission.^[^ ^46^ ^]^ Copyright 2020, Wiley‐VCH.

Following this aptitude of matrix modification, another work in water treatment technologies focused on synthesizing a GFM material with —C=N— bonds in its framework.^[^ ^46^ ^]^ Specifically, Chen et al. fabricated a triazine‐based GFM which can be considered as an N‐doped GY (**Figure** **5**), herein designated as TA‐GY.^[^ ^46^ ^]^ The catalyst synthesis was carried out by reacting cyanuric chloride and dilithium salt of 1,4diethynylbenzene, achieving a 75% yield of TA‐GY. Due to the *s*‐triazine composition of the cyanuric chloride precursor, the resulting material shows a topologically similar structure to *γ*‐GY due to its diacetylenic bonds and unitary cell, being labeled as a graphyne analogue. However, the high *π*‐electron conjugation (due to the carbon‐nitrogen bonds) endowed the semiconductor with a more facile electron‐hole generation. The CB and VB potentials, obtained by DFT calculations, are respectively 4.50 and −5.94 eV (vs vacuum), with a narrow bandgap energy of 1.44 eV. This allowed for visible light activation, with 99% MO removal in 8 h and 100% elimination of *Escherichia coli* after 1 h of irradiation being achieved (Table 1). This work prompted to show that GFM can easily take part in a broader scope of photocatalytic applications since it proved to be efficient in disinfection.

### 2.2 Production of Hydrogen

Hydrogen production is widely considered a next generation technology for energy conversion and an alternative to fossil fuels since several sustainable sources can obtain it, such as biomass or water.^[^ ^83^ ^]^ The synthesis via photocatalytic water splitting is an attractive route that has shown increasing global attention.^[^ ^84^ ^]^

Lv et al. synthesized a GDY/CdS heterojunction and performed photocatalytic hydrogen evolution studies with LED radiation.^[^ ^37^ ^]^ GDY was prepared by growth on a copper foil and contacted with cadmium and sulfide sources using a solvothermal method.^[^ ^37^ ^]^ Under the same experimental conditions, GDY alone was not capable of producing any H_2_. Increasing amounts of CdS present on the GDY surface allowed for the more significant generation of H_2_. The best performing heterojunction allowed for a 2.6‐fold improvement in comparison to CdS alone (Table 1). This change is due to the high carrier mobility and hindered exciton recombination. The band structure can be determined through Mott‐Schottky plots and X‐ray photoelectron spectroscopy to justify the water splitting mechanism, by showing GDY has slightly less positive band levels than CdS leading to higher availability of electrons in the CdS moiety. Moreover, light absorption was also enhanced on the composite and further allowed to promote H_2_ evolution owed to the more facile photoactivation.

**Figure 6:**
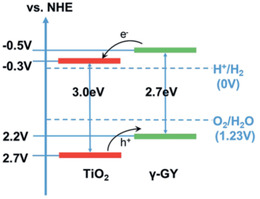
Band structure of GY/TiO_2_ composite. Reproduced with permission.^[^ ^38^ ^]^ Copyright 2018, Royal Society of Chemistry.

Wu *et al*. fabricated a GY material by a mechanochemical approach and combined it with TiO_2_ through a hydrothermal method to produce a composite.^[^ ^38^ ^]^ This synthesis route was proven to lead to the production of a GY material with p‐type semiconducting ability, exhibiting visible light absorption ability.^[^ ^24^ ^]^ The composites with higher amounts of GY presented higher visible light absorption and reduced photoluminescence, which are in general considered as promising characteristics for efficient photoactivity. The photocatalytic evolution of H_2_ was performed in a 9:1 water:methanol solution and the composite achieved an 8.4 times higher amount of H_2_ in 4 h when compared to pure TiO_2_ (*1*). The band structure was determined by the Mott‐Schottky plots (**Figure** **6**). The enhanced mechanism is explained by the VB of GY being less positive than that of TiO_2_. This allows for holes to be transferred to GY and enables a higher availability of electrons in the CB of TiO_2_, more capable of inducing H_2_ formation.

The combination of GDY with another carbon material is an exciting option for the development of metal‐free photocatalysts. Xu et al. prepared a composite consisting of GDY synthesized by the typical Glaser reaction on a copper substrate and graphitic carbon nitride (GCN) obtained by a calcination method.^[^ ^39^ ^]^ X‐ray powder diffraction patterns show that the individual crystal structures of both components are maintained in the composite. A decrease in the intensity of the bands corresponding to amino groups of GCN in Fourier‐transform infrared spectra was observed, owing to the reaction of these groups with the butadiyne bridges of GDY.

**Figure 7:**
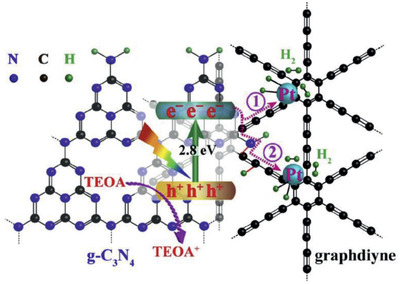
Photocatalytic mechanism routes and band structure of GDY/GCN with Pt nanoparticles. Reproduced with permission.^[^ ^39^ ^]^ Copyright 2020, Elsevier.

The composite showed a 4.5‐fold enhancement on photocurrent intensity, i.e., higher charge separation efficiency, and electrochemical impedance spectra lower arc radius, demonstrating reduced charge transfer resistance. The time‐resolved fluorescence spectra confirmed the improved composite photoactivity by providing a prolonged lifetime of charge carriers. The band structure was determined with a 2.8 eV bandgap and a −0.68 eV flat band potential (vs NHE), which is appropriate for H_2_ evolution. Moreover, since GCN shows a smaller work function that GDY, the electrons from GCN are easily transferred to GDY, which are then transferred to platinum deposited on the surface of GDY, which acts as co‐catalyst to promote H_2_ evolution. Triethanolamine was also included in the photocatalytic system to provide an oxidation reaction and guarantee the separation of electron/hole pairs. The GDY/GCN catalyst shows two routes for H_2_ evolution: electrons from the CB of GCN migrate through GDY to Pt i) by the butadiyne bridges and ii) by the newly formed CN bonds, as depicted on **Figure** **7**. These features produced a nearly 7 times higher H_2_ formation rate when compared to bare GCN (Table 1). Also, compared to other carbonaceous materials (carbon nanotubes, carbon fibers and graphene), this of GDY/GCN shows considerably higher efficiencies for H_2_ evolution according to the authors.^[^ ^39^ ^]^

Schwarz et al. prepared triazine‐based GDY materials by two methods: i) the described Glaser coupling reaction on a copper substrate (GDY‐G); and ii) following the Sonogashira protocol which employs a platinum‐copper catalyst (GDY‐S).^[^ ^73^, ^85^, ^86^ ^]^ Both reactions resulted in butadiyne linked triazine GDY polymers; however, GDY‐G maintains a higher crystallinity (with some unreacted C≡C—H ending groups), and GDY‐S shows an apparent amorphous structure. The GDYG material enables efficient charge transfer, but the disordered GDY‐S promotes the blockage of recombination, which benefits photocatalytic activity. Under visible light irradiation, with triethanolamine as sacrificial donor and platinum as co‐catalyst, the H_2_ evolution rate was 2 times higher when employing the amorphous material (GDY‐S) compared to the ordered one (Table 1).

Very recently, a study using a GDY/CuI hybrid photocatalyst was published for H_2_ evolution.^[^ ^40^ ^]^ The synthesis method differs from the typical polymerization reaction on a copper substrate since it employs a cuprous iodide (CuI) co‐catalyst to promote GDY growth. For this reason, the resulting material is a GDY/CuI hybrid. The presence of copper ions during the cross‐coupling reaction effectively enhanced the photoactivity of GDY. The photocatalytic experiments were performed in the presence of triethanolamine as a sacrificial agent and Pt as co‐catalyst. Electrons migrated from GDY/CuI to Pt, promoting H_2_ formation. X‐ray photoelectron spectroscopy analysis showed that after Pt loading, there was a shift on the binding energies proving the effective electron transfer from GDY/CuI to Pt which is beneficial for H_2_ formation. Transient fluorescence dynamics was investigated with eosin yellow dye, and it was observed that the dye's excited state had a much longer lifetime in the presence of GDY/CuI than with GDY only (0.60 ns compared to 0.25 ns, respectively). Photoelectrochemical measurements also demonstrate that the anchoring of CuI in GDY is beneficial for the photocatalytic activity since the hybrid material shows a higher photocurrent generation (i.e., a faster electronic transfer) and a more efficient charge separation observed by linear sweep voltammetry. The photocatalytic results demonstrate a 15.8 times higher rate of H_2_ formation with GDY/CuI than with GDY and a 3.0 times increase in comparison with CuI alone (Table 1).

Recently, Wu et al. published a ternary heterojunction comprised of TiO_2_, MoSe_2_, and GY (MoSe_2_/TiO_2_/GY).^[^ ^70^ ^]^ In this work, GY was prepared by a mechanochemical route and combined with MoSe_2_/TiO_2_ nanosheets by a hydrothermal method.^[^ ^25^ ^]^ The dosages of each moiety were varied and tested, with the best H_2_ production activities being achieved using a TiO_2_ photocatalyst loaded with 0.1% MoSe_2_ and 2.5% GY. This heterojunction, in the presence of a 10% methanol solution and a Xe lamp radiation source, leads to an H_2_ production rate of 800 μmol h^1^ g^–1^. The authors compared this evolution rate with TiO_2_/GY, TiO_2_/MoSe_2_ and TiO_2_ alone (Table 1). The intensity of photoluminescence measurements showed that the final heterojunction was the lowest represented a hindered recombination of charge carriers. Moreover, the electrochemical impedance spectra revealed a smaller internal resistance to charge transfer in the ternary material. These characterizations were fundamental to justify improvement in H_2_ production.

**Figure 8:**
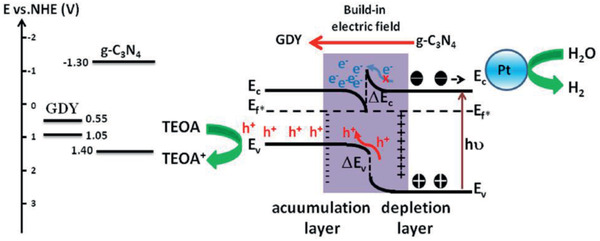
Band structure and charge transfer mechanism in GCN/GDY material. Reproduced with permission.^[^ ^67^ ^]^ Copyright 2020, Elsevier.

Si et al. reported a heterojunction for H_2_ production, based on a GCN/GDY system.^[^ ^71^ ^]^ GDY was prepared by a cross‐coupling method, and the heterojunction prepared by a solvothermal method. This material showed prominent characteristics due to its band structure and by the aid of platinum as co‐catalyst (**Figure** **8**). The highest H_2_ production rate was achieved for the material with 1% GDY in the GCN matrix and was up to 22 712 μmol h^‐1^ g^–1^ in the optimized system using 15% (v/v) TEOA aqueous solution with H_2_PtCl_6_ added as a co‐catalyst. The improved performance (3 times higher evolution rate) compared with sole GCN (Table 1) is discussed in terms of the charge separation tests. Electrochemical impedance spectroscopy and photocurrent measurements demonstrate the improved electron‐hole separation and faster electronic transfer by an intrinsic generated electric field between GCN and GDY, depicted in Figure 8. Then, photoluminescence spectroscopy showed suppression of the recombination process. Furthermore, by last, time‐resolved fluorescence decay spectroscopy showed that the lifetime of charge carriers was ultimately extended, being experimentally proven to rise to 42%.

**Figure 9:**
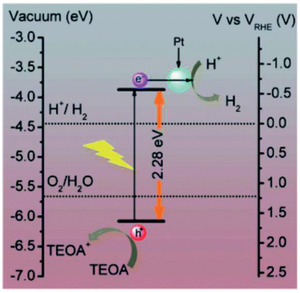
Energy levels of DBA‐GDY and semireactions occurring on each band in the presence of triethanolamine (TEOA) and Pt. Reproduced with permission.^[^ ^69^ ^]^ Copyright 2020, Royal Society of Chemistry.

Shen et al. reported a 3D GDY‐based material for H_2_ formation.^[^ ^74^ ^]^ The synthesis protocol consisted of selective Sonogashira and Glaser coupling reactions (which were abovementioned) with CuCl as the catalyst to yield a dehydrobenzoannulene polymeric system (DBA‐GDY), which is structurally similar to GDY and considered as a 3D GFM.^[^ ^85^, ^86^ ^]^ Through the Tauc plot and by cyclic voltammetry, the bandgap was calculated to be 2.28 eV with the corresponding VB and CB energy levels as −6.12 and −3.84 eV (vs RHE), which are sufficient for H_2_O reduction to H_2_ and H_2_O oxidation to O_2_, as depicted in **Figure** **9**. The photocatalytic experiments were performed using triethanolamine as a sacrificial agent and Pt as co‐catalyst to improve the H_2_ generation, up to 340 μmol h^–1^ g^–1^ under visible light irradiation (Table 1). DBA‐GDY achieved a relatively efficient H_2_ generation without combination with another material. This work opens a synthetic route to develop new 3D GFMs in order to promote efficient photocatalysis. This transition into 3D GFMs has been briefly mentioned in a previous mini‐review, in which it is highlighted the unique morphology of DBA‐GDY to achieve an effective photocatalytic water splitting method.^[^ ^68^ ^]^

For the hydrogen evolution reaction, GFMs showed a critical role in balancing the distribution of charge carriers. The main aspect of retaining from the available experimental research is that photoelectrochemical measurements corroborate that GFMs contributed to an easier formation of hydrogen, with or without sacrificial agents. This was due to the electron scattering effect that GFMs promoted, allowing for heterostructures with suitable bandgap energies and properly positioned conduction band edges.

### 2.3 O_2_ Evolution via Water Splitting

The production of O_2_ via water splitting photocatalytic process has been investigated by many authors using several materials, sacrificial agents and light sources mainly due to the simplicity and good performance of photocatalysis.^[^ ^84^ ^]^

**Figure 10:**
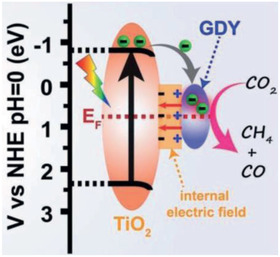
Scheme of the TiO_2_/GDY heterojunction with an internal electric field‐induced charge transfer and separation under light irradiation for CO_2_ photoreduction. Reproduced with permission.^[^ ^36^ ^]^ Copyright 2019, Wiley‐VCH.

Si et al. synthesized a composite consisting of GDY, GCN, and APO.^[^ ^33^ ^]^ APO alone is established as an efficient benchmark photocatalyst for O_2_ evolution due to its high photoactivity, proven through DFT calculations and justified by its electronic band structure and redox power.^[^ ^87^ ^]^ GCN is a promising photocatalyst for a variety of applications, and the efficacy of a GCN/APO for the production of O_2_ has been previously demonstrated.^[^ ^88^ ^]^ The GDY/GCN/APO material was prepared by self‐assembly after stirring the different initial materials in suspensions, with GDY being fabricated on a copper substrate by a cross‐coupling reaction.^[^ ^33^ ^]^ The composite was irradiated with visible light in an aqueous solution, and the O_2_ formation rates were 12.2 times higher than that of assays performed with APO (Table 1). This improvement is ascribed to the effective Z‐scheme with a strong interface interaction. The interaction between the co‐catalysts allowed for a higher photocurrent and fast charge separation and transfer. The photogenerated electrons are transferred from APO to GDY and subsequently trap holes from GCN, and the latter can be transferred to the GDY due to high carrier mobility (**Figure** **10**). This mechanism allows for a superior availability of holes on APO which have strong oxidizing power and improve water splitting.

**Figure 11:**
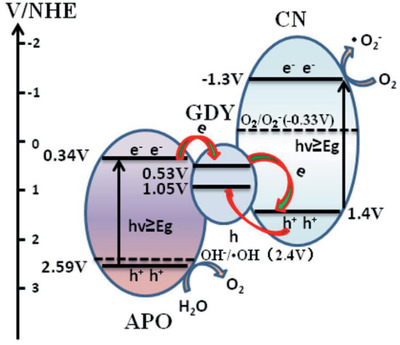
Proposed electron transfer mechanisms of the APO/GDY/GCN Z‐scheme system. Reproduced with permission.^[^ ^33^ ^]^ Copyright 2018, Elsevier.

As discussed in more detail in Section 2.1, Guo et al. prepared a Pickering APO/GDY emulsion and tested it for MB removal and O_2_ evolution.^[^ ^34^ ^]^ This group investigated the combination of APO with different carbon materials, and the APO/GDY emulsion resulted in the highest apparent rate constant for MB degradation. When tested for O_2_ evolution, the system allowed for the water oxidation redox reaction to occur with GDY acting as an electron acceptor due to its VB being located within the bandgap of APO. The mechanism of both MB and water oxidation consists of the photogeneration of charges transferred from the CB of APO to the VB of GDY (Figure 11). This enhanced charge transfer is attributed mainly to the *π*‐conjugation in the GDY structure.

Moreover, GDY allows for electroless deposition of some metal ions, and in the case of APO, can help to combat the photocorrosion. Guo et al. performed X‐ray photoelectron spectroscopy to verify this and concluded that Ag^+^ was reduced to Ag^0^, hindering the corrosion process and improving the stability of the emulsion. This phenomenon was not observed with the other tested carbon materials and is probably the reason why GDY performs more efficiently.

As specified before, the higher photocatalytic activity is owed to the carbon acetylenic linkages promoting a strong attachment with metallic frameworks, enhancing charge transfer and accelerating the photocatalytic redox reactions.

**Figure 12:**
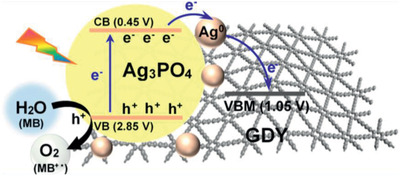
Proposed mechanism for the photocatalytic reactions with the APO/GDY composite under visible light irradiation. Reproduced with permission.^[^ ^62^ ^]^ Copyright 2019, American Chemical Society.

Cellular hypoxia is a real concern caused by tumors, and its treatment has been systematically investigated by applying photodynamic therapy with photocatalysts capable of evolving oxygen and producing reactive oxygen species (ROS).^[^ ^89^, ^90^, ^91^ ^]^ Jiang et al. proposed GDY oxide (GDYO) nanosheets as a biomimetic material for water oxidation.^[^ ^35^ ^]^ GDY was synthesized by a cross‐coupling reaction on a copper substrate, and its oxidation was performed by acid treatment and ultrasound‐mediated exfoliation. By Raman analysis, it was detected that the GDY structure is partially affected by the oxidation process. The GDYO nanosheets were used for the oxygen photocatalytic production studies in the presence of AgNO_3_ as the sacrificial agent and under visible light irradiation (**Figure** **12**), achieving a 31.4 improvement factor comparing with the nonoxidized material (Table 1). The valence band energy level of GDYO favored oxygen production since it is slightly more positive (Figure 12), and photoelectrochemical measurements show that a higher photocurrent response is reported for GDYO compared to the initial material.

After that, the GDYO nanosheets were anchored on a red blood cell membrane for reoxygenation of the hypoxic cells with a 660 nm laser in tumor‐bearing mice. Biocompatibility tests were performed before‐hand. After the photodynamic therapy tests, it was revealed a hypoxia relief, allowing for cell reoxygenation and the suppression of tumor growth. The antitumor performance of GDYO anchored on red blood cell membranes was proven to be effective with this study and showed a novel process employing a biocompatible photocatalyst.

The publication by Jiang et al. reveals the first usage of GDYO for photocatalysis.^[^ ^35^ ^]^ It is interesting to mention that both GDY and its oxidized form had been previously used as substrates for electroless metal deposition and in electrochemical studies due to their reducing power and low work function.^[^ ^54^, ^92^ ^]^ So, GFMs are promising metal‐free materials with high photoactivities, i.e., without combination with a metal‐based semiconductor.

### 2.4 Greenhouse Gases Mitigation

**Figure 13:**
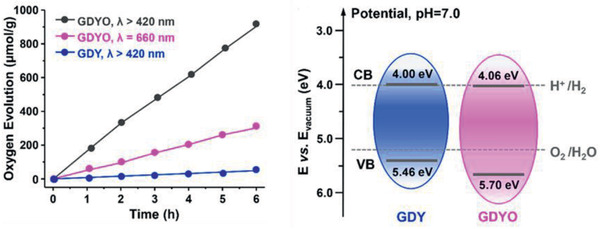
a) Evolution of oxygen over time with GDY and GDYO under visible light (*λ* > 420 nm) and laser (*λ* = 660 nm) irradiation and b) electronic band structure of GDY and GDYO. Reproduced with permission.^[^ ^35^ ^]^ Copyright 2019, American Chemical Society.

The photocatalytic reduction of carbon dioxide (CO_2_) is a potential solution to overcome the increasing emissions of greenhouse gases. Moreover, besides CO_2_ reduction, there is a concomitant water splitting reaction that can induce O_2_ generation. For instance, Xu et al. prepared a TiO_2_/GDY hybrid photocatalyst for the reduction of CO_2_, generating O_2_ besides methane and carbon monoxide.^[^ ^36^ ^]^ The material was synthesized by an electrostatic self‐assembly procedure and further calcination, with the initial GDY obtained by a cross‐coupling reaction on a copper surface. It was observed that the photoreduction was highly dependent on the adsorption of CO_2_ molecules on the catalyst. During photocatalysis, the electrons of TiO_2_ are transferred to GDY by the internal electric field that is created between TiO_2_ and GDY as depicted in **Figure** **13**. The electron‐rich GDY is the major contributor to the photoreduction; the active sites were found to be the acetylenic bonds in GDY. The photocurrent density was much higher on the hybrid photocatalyst than over TiO_2_ alone and explained a slower recombination of electron‐hole pairs. Also, electrochemical impedance spectroscopy showed that the hybrid catalyst has superior charge transfer enabling for a higher photoactivity. Simultaneously to CO_2_ reduction, the subproduct O_2_ formation was followed registering a rate of 30.9 μmol g^–1^ h^–1^, which is ≈3.7 times higher than when using only TiO_2_, i.e., GDY acts as an efficient co‐catalyst (Table 1).

Cao et al. synthesized a CdS/GDY and a CdS/GO (GO—graphene oxide) materials to essentially compare the charge transfer obtained after photochemical activation and provide proof that GFMs are more efficient as co‐catalysts than GRDs.^[^ ^69^ ^]^ Their main application was the study of CO_2_ removal. GDY was synthesized by a cross‐coupling reaction on a copper substrate and GO by a modified Hummer's method.^[^ ^12^, ^93^ ^]^ The composites were obtained by an ultrasonic treatment followed by a solvothermal with cadmium acetate. Both materials were employed in the same gas‐phase CO_2_ reduction system. The CO_2_ conversion yields (Table 1) were increased from 15.0 to 18.7 μmol g^‐1^ h^–1^ (a 25% improvement rate from using GDY to GO). The authors discuss the improved activity of the GFM‐based catalyst based on four main causes: i) higher CO_2_ adsorption performance of GDY, quantified to be 4x times higher than using CdS/GO, and ascribed to the empirically observed hollow locations and vacancies in the composite surface; ii) slower recombination proved by the decayed luminescence lifetimes in the defect‐induced electron trapping sites; iii) more efficient interfacial charge carrier transfer, sustained by DFT calculations and electrochemical measurements; and finally iv) stability over reuse tests due to the stronger interaction between GDY and CdS. This work provides both theoretical and experimental insights on why GFMs can be a more promising carbon material in photocatalytic applications than GRDs.

### 2.5 NADH Regeneration System

The redox couple NAD^+^/NADH is pivotal in the metabolism pathway for carrying electrons between redox reactions during cellular respiration and are found in all forms of life. NAD^+^ is automatically regenerated to NADH by naturally occurring enzymatic reactions. The artificial mimicking of this process can be performed using the well‐established photocatalytic system with an electron mediator ([Cp*Rh(bpy)(H)]^+^) and triethanolamine as a sacrificial electron donor.^[^ ^94^ ^]^ Pan et al. synthesized nitrogen‐doped GDY nanosheets for NADH regeneration, reporting that few studies focus on metal‐free photocatalysts for this application.^[^ ^32^ ^]^ They attested that nitrogen‐doped carbon materials are a promising technology for several research topics, e.g., N‐GDY as an effective electrocatalyst for oxygen reduction.^[^ ^95^ ^]^

**Figure 14:**
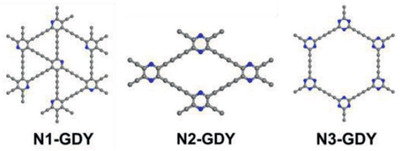
Proposed structure of N1‐GDY, N2‐GDY, and N3‐GDY. Reproduced with permission.^[^ ^61^ ^]^ Copyright 2019, American Chemical Society.

Additionally, computational predictions show that nitrogen‐doping facilitates charge transfer and promotes oxygen reduction by allocating positive charges to the carbon atoms neighboring nitrogen atoms.^[^ ^96^ ^]^ Pan et al. fabricated three different materials by polymerization of different precursors. These materials were prepared by a liquid/liquid interface between an organic phase comprised of the precursor (triazine, pyrazine, and pyridine monomers) dissolved in tetra‐*n*‐butylammonium fluoride and a cupric ion (Cu^2+^) aqueous mixture. After removal of both phases, the resultant material was denoted according to the precursor (N1‐GDY, N2‐GDY, or N3GDY, respectively; **Figure** **14**). The polymerization of these monomers leads to different nitrogen contents, configurations and distribution of pores. In this way, the authors reported a fine control of the composition and structure of GDY, yielding N1‐GDY, N2‐GDY, and N3‐GDY with increasing nitrogen content, corresponding to triazine, pyrazine and pyridine monomers (Figure 14).

The photocatalytic assays with these three materials allowed to understand that a higher nitrogen content on GDY increases the NADH regeneration performance since the material was more hydrophilic and the aqueous solution had a facilitated contact with the catalyst surface. Through electrochemical analyses, the flat‐band potentials were determined, and it was concluded that a more negative conduction band is beneficial for improved NADH photocatalytic regeneration. Pan et al. defend that, despite not showing a high yield compared to the literature, the obtained results are still very promising accounting for the metal‐free advantages of their synthesized materials.^[^ ^32^ ^]^ Yet again, the easy alteration through the synthesis route permitted to obtain GFM optical semiconductors with different characteristics, the controlled architecture with acetylenic linkages and carbon‐nitrogen aromatic bonds accelerating charge transfer and promoting redox reactions in the system.

## 3 Summary and Outlook

The first experimental photocatalytic study of a GFM material was published in 2012. However, theoretical studies represent the most significant share of literature. The as‐prepared materials are not in pristine conditions, and predicted properties differ from the experimental ones since considerations such as one‐atom‐thickness and total crystallinity are distant from reality. In 2010, the first GFM material was fabricated, and ever since, the research interest in the application of these materials has steadily increased. There have been tremendous efforts to achieve greater efficiency, structure control, scalability, and sustainability.

Herein, we have summarized the most recent progress in the synthesis of GFM‐based materials for photocatalytic applications. Nearly all the reviewed publications preferred a wet chemistry method using hexaethynylbenzene diluted in different solvents with a copper substrate to grow GDY or GY (the typical Glaser coupling reaction) — only three studies applying a mechanochemical route, by combining calcium carbide and hexabromobenzene. Additionally, the combination with other materials was seldomly metal‐free (only two studies with GCN) and merely a few others reported modifications which did not involve metals, such as N‐doping or oxidation. The remaining synthesis routes concerned different structures being constructed, such as *β*‐GDY, a 3D GDY or an amorphous GDY. It is interesting to note that the highest improvement factors were verified when the ultrasonic oxidation was performed, generating an oxidized form of GDY (GDYO). Despite that metal oxides composites closely follow GDYO, the few combinations with carbon materials (GCN and GO) also presented high improvement factors. One particular study focused on comparing the use of GRDs and GFMs to test which could be a better catalyst, specifically for CO_2_ reduction, and GFMs proved their worth by excelling in electronic transfer and charge carrier lifetime.

This review also encompasses the analysis on the effects of each modification step in the original GFM. Overall, these modifications were performed to reduce the natural hydrophobicity of GFMs and increase the photoactivity, by tweaking the band structure, hindering carrier recombination and enabling faster charge transfer. In most of the publications, the charge carrier mobility was investigated and optimized for higher electron transfer to promote specific redox reactions and increase productivity. Therefore, the treatments performed in the selected studies provide insights into the efficient construction of GFMs for specific catalytic photoconversion processes. The collection of these papers shows different synthetic paths and offers insight into how to efficiently produce hybrid materials. Applying the hydrothermal or solvothermal methodologies, being the most widely employed, resulted in successfully photoactive composites. However, different procedures may be applied for superior material engineering. For instance, GFMs could be combined with other materials by cross‐linking, precipitation or annealing techniques depending on the desired characteristics and aimed application.

The potentialities that GFMs demonstrate could be further applied in a broader range of photocatalytic functions, such as H_2_O_2_ production, removal of air pollutants, N_2_ fixation or biomass conversion. Particularly, owing to the current environmental concerns, the carbon dioxide reduction reaction can be exploited further through the combination of electronically suitable GFMs and specific photocatalysts already used for this application, such as non‐noble metal semiconductors. Furthermore, due to their metal‐free nature, GFMs are a promising group of materials for biocatalytic applications, as described in the NADH regeneration study, establishing new routes for the treatment and diagnosis of health issues, through mediated drug delivery, biosensorial detection of infectious diseases or even photodynamic therapy. We can also expect a proficient return when employing GFMs in other systems ascribed to both expected and demonstrated properties. For instance, adsorptive and thermal capabilities could be explored for catalyzing chemical reactions.

We hope that this compilation of studies will assist in developing efficient photocatalytic systems based on GFMs, as there is still plenty of room for creating more sustainable synthetic routes, developing superior photoactivity and start modeling novel systems for new and yet unforeseen applications.

## Conflict of Interest

The authors declare no conflict of interest.
